# SFD for ocean conservation—assessing the development of ocean conservation related competences through a sport for development intervention amongst adolescents in Cape Town, South Africa

**DOI:** 10.3389/fspor.2026.1638238

**Published:** 2026-05-29

**Authors:** Sally-Ann Jennifer Fischer, Simona Safarikova

**Affiliations:** 1 Institute of European Sport Development and Leisure Studies, German Sport University, Cologne, Germany; 2 Department of Development and Environmental Studies, Faculty of Science, Palacký University Olomouc, Olomouc, Czechia

**Keywords:** Sport for Development, competence development, conceptual framework, environmental protection, ocean conservation

## Abstract

**Introduction:**

The Sport for Development (SFD) sector has so far enjoyed a longstanding practice in tackling socio-economic challenges in South Africa. Despite the increasing catalogue of topics addressed in SFD, the uptake of environmental protection, and further ocean conservation, is still uncommon and only practiced by a small number of local NGOs. Considering that the ocean's resources are indispensable to the survival of South Africa's population, this research aimed to address the mentioned topic within SFD (and in South Africa) by developing an SFD conceptual framework that teaches young adolescents ocean conservation and competences related to it in Cape Town, South Africa.

**Methodology:**

Based on the experiential learning theory by David Kolb, the conceptual framework includes a specifically developed ocean conservation competence framework, conveyed by carefully adapted sport-based activities. This SFD intervention was deemed as complimentary to the Ocean Guardian Workshop (OGW) of the collaborating nongovernmental organization I AM WATER. During the OGW, young adolescents are taught how to snorkel in the ocean, giving them the opportunity to physically experience the ocean, while learning and developing a sense of embodiment and emotion towards preserving it. Aiming at deepening this impact, the SFD intervention was applied directly after the completion of the OGW for four consecutive weeks. To evaluate this intervention, a quantitative pre and post- survey approach was applied, addressing the ocean conservation related competences acquired throughout the combined OGW and SFD intervention.

**Results:**

The results were analyzed using an independent t-test and demonstrate no significant differences regarding the development of ocean conservation related competences.

**Discussion:**

The discussion places great emphasis on the reflexivity of the researcher, highlighting the short-term nature of the study and the dynamics in Global North to Global South research.

## Introduction

Under the term “sport-for-development” (SFD)^1^, sport has been established within development cooperation as an instrument designed to tackle a variety of development issues. In fact, while the number of global challenges is continuously rising, so is the catalogue of topics in which sport supposedly has a positive impact (1, 5–8). With an ever-growing mythopoeic status, as Coalter (9) describes it, there is an underlying assumption that sports ability to tackle issues beyond the personal development of individual character and values has no boundaries. This trend was only intensified in 2015 when the United Nations (UN) officially recognized sport as an enabler for several of the 2030 Agenda Sustainable Development Goals (SDGs). Ever since, SFD organizations have increased their scope of objectives and designed their projects to focus on wider socio-economic concerns such as physical health, gender equality, or employability (1), and most recently environmental protection (10–12). A vast number of scholars have already criticized this development in the past, pointing towards an unrealistic proportion of responsibility placed upon sport to solve existential threats (13–16). Particularly concerning the topic of environmental protection, it seems that the SFD sector is being driven to take on climate action into their repertoire of programs (10). While anthropogenic pressures continue to exacerbate our climate, it is undoubtedly necessary for all economic sectors to increase their climate action measures (54). However, the aim of this article is to assess the impact of a SFD program on successfully teaching competences related to ocean conservation to the people it serves. To do so, this article presents a specifically developed SFD conceptual framework that includes a SFD pilot program focused on teaching ocean conservation competences to young adolescents in Cape Town, South Africa, while discussing its limits and shortcomings. Additionally, considering that the researcher is from Europe and is implementing an SFD program in South Africa, this article aims at analyzing the collected data with increased researcher reflexivity throughout the discussion.

While the SFD sector has so far been employed as a longstanding practice to tackle socio-economic challenges in South Africa, the uptake of environmental protection, and further ocean conservation, is still uncommon and only practiced by a small number of local NGOs (1, 2; 56). However, particularly within South Africa, socio-economic prosperity is very much tied to environmental and especially ocean related health. Besides the general benefits of the ocean such as providing drinking water, producing over 50% of oxygen and storing over 90% heat since the industrial revolution (17, 18), South Africa's communities are considered as subsistence fishers, relying heavily on marine resources for food and economic security (3, 19). The Atlantic and the Indian Ocean surrounding South Africa come with an abundance of fish species and populations, providing the country with an immense nutritional value. Additionally, large- and small-scale fisheries, and the mining and tourism industries are vital resources for job creation (20). Therefore, increasing anthropogenic pressures on the climate are having adverse effects on the livelihoods of South Africa's population, increasing calls for climate action measures to be taken. Protecting the ocean is paramount as it ultimately implicates human survival. As a result, there is a need to sensitize the communities to the dangers inherent to human's careless behavior to foster ocean protection through ocean friendly activities. This is where SFD has been increasingly brought into focus.

The trend towards the uptake of climate action in the SFD sector is also apparent in recent literature. However, ocean conservation remains a particular niche, as current studies are predominantly focused on issues such as land degradation and pollution, and the sector overall (21, 57). Giulianotti et al. (10) pioneered this area and highlighted how the sector generally is struggling to take on climate action due to other more immediate threats experienced within their communities. In fact, Soares Moura and Scott (2) point towards unclear global macro policies that are difficult to translate to local specificities and therefore lack appropriate guidance for SFD NGOs to take on climate action measures. Additionally, climate change is viewed as extremely complex, presenting inconsistencies in approaches from macro to micro levels especially within the development sector, as highlighted by Ali et al. (22). In a world that increasingly requires global governance, so called glocalization is of utmost importance to provide local NGOs with adequate support. A holistic approach is needed, streamlining climate change within the entire sector and organizational culture of SFD NGOs (21). While Giulianotti (14) proposes a socio-ecological approach to bridge this gap, giving a clear outline of how climate action should be ingrained into SFD organizations, there is no understanding of how far climate action measures taken within SFD can translate from the sector into the communities they serve. This is where the following study will add a vital contribution, investigating how a SFD program can effectively transfer necessary ocean conservation measures into life skills, transferring these into the daily life of the targeted communities.

Considering the difficulties to take on climate action and the scarce literature thereof, it is no surprise that the availability of data concerning ocean conservation is practically non-existent in the realm of SFD. Ocean conservation, also known as marine conservation, encompasses the protection and perseveration of marine resources, while preventing further exploitation through responsible and sustainable usage (23). Generally, marine based sports have been identified as particularly useful in creating experiences that lead to greater place attachment and consequently greater initiatives to protect these places. As Olive (24) found, the ocean has an incredibly tangible nature, putting the participant throughout the activity directly into contact with the challenges the ocean is facing. While currently there is no clear indication of water sport athletes acting more environmentally or ocean friendly (25), the immersive experience suggests that participants create a bond with the ocean and are therefore more inclined to preserve it. Evidence by Lucrezi and du Plessis (26) supports these findings, especially in the South African kelp forest, further alluding to the idea that SFD could be a useful method to teach ocean conservation practices. Nevertheless, it is important to note that previous studies have focused on participants who already engage in marine based sports, while the SFD sector should be concerned with teaching ocean conservation to marginalized communities. Therefore, the idea to engage in ocean conservation practices requires the SFD sector to increase ocean literacy in those communities overall. To be considered an ocean literate person, one must understand the importance of the ocean towards humankind, be able to communicate about it to others and be able to make informed decisions that are ocean friendly, recognizing one's own responsibility (27). Additionally, according to McKinley and Burdon (28), there must be an experiential component, in which one has direct experiences with the ocean to trigger behavioral changes and make those informed decisions (53). Increasing ocean literacy with active experiences therefore constitutes the basis on which the research in this article builds on. Particularly, the factor of the experience is an important distinction, as it draws on the fundamental idea of SFD, creating scenarios during sport activities that teach participants how to behave in other life contexts. This concept is based on the Experiential Learning Theory by David Kolb (4). Kolb (4) suggests that learning takes place in a cycle of four stages: the concrete experience, the reflective observation, the abstract conceptualization, and finally the active experimentation. The participants are confronted with an experience, then reflect on it, they continue to interpret what they experienced within the given context, and lastly, put their newly acquired knowledge into practice. The underlying idea is that participants who engage with the experiential learning cycle develop competences that are applicable not only within sport activities, but more meaningfully, in real-life contexts relevant to ocean conservation. The sport itself provides modes of physical activities and an environment in which challenges are often inherent and need to be overcome to attain certain goals. Therefore, sport can host related scenarios in a more friendly and less intimidating manner, leaving room for the participants to learn and adapt behavior or even try out new ways of dealing with conflict. The following study is building on the previous literature, using the experiential approach and exploring whether ocean literacy can also be transferred on a playing field rather than in the ocean. The study is therefore expanding previous research, circumventing the need to engage physically in water-based sports while still providing an embodied experience on the ground.

Inherent to SFD and highlighted in literature presented above, the embodied experience is crucial to develop ocean conservation related behavior (11, 24, 26). As there are assumptions that SFD will be able to develop such a behavior, this study has created a SFD conceptual framework that is designed to increase young adolescent's ocean related knowledge and strengthen competences in relation to ocean conservation.

## Methodology and concept development

The SFD conceptual framework fundamental to this research, has been developed in close collaboration with the NGO called I AM WATER (IAW). IAW has operated in Cape Town, South Africa, since 2010, and focuses on teaching young adolescents how to engage in ocean conservation practices (52). Under their flagship program Ocean Guardian Workshop (OGW), young adolescents from South African primary schools learn how to snorkel in the ocean, creating a transformative embodied experience where the participants are immersed into the environment and develop a sense of personal attachment to it. IAW is one of the very few SFD NGOs that utilizes physical activity to teach ocean conservation competences and makes use of the exciting experience of snorkeling to facilitate behavioral change, as suggested by Kolb's Experiential Learning Theory (4). The SFD conceptual framework was developed as an extension to the OGW, closely adapted to its objectives and content. Taking place on the participants school ground, the SFD conceptual framework was designed to use other forms of physical activities to increase the participants' knowledge about the ocean and further reinforce ocean conservation related competences which they have already touched upon during the OGW. The conceptual framework acted as a medium to bring this new subject area into the daily environment of the participants. Following the SFD concept of the German Agency of International Cooperation (GIZ) and the German Sport University (GSU)^2^, the conceptual framework is comprised of two components. For one, it was concerned with the identification of ocean conservation related competences, and secondly, the development and delivery of adapted SFD training sessions targeting those competences as well as knowledge retention.

### Research design

To identify whether the SFD conceptual framework was able to fulfil its purpose to foster ocean conservation related competences amongst young adolescents, a quantitative research design was applied, administering surveys amongst the participants that were statistically analyzed. Given the rigorous characteristics of quantitative research, this study aimed at generating highly objective and unbiased results which can therefore be easily reproduced and generalized to the wider target population (29).

### Data collection

The study sample was chosen via convenience sampling as it included the school students that participated in the OGW of the NGO IAW. Therefore, the sample consisted of students from a public primary school in Cape Town, South Africa, representing predominantly black and colored communities from the surrounding area. The group was mixed in gender and averaged 13 years of age. The students were divided into two groups during the OGW and remained within these groups for the additional SFD training sessions focused on ocean conservation competences. Each group consisted of 36 participants (72 in total), conforming to the Safeguarding Practitioner Guideline^3^ (51).

Data was collected via pre and post surveys. The pre-survey was completed by the participants a few days before they took part at the IAW OGW, while the post-surveywas completed one week after they completed the additional SFD training sessions. The survey included 36 items, of which one part was directed towards the retention of ocean related knowledge and the other targeting the ability to recognize ocean conservation related competences. A mix of closed and open questions were applied to avoid survey fatigue. Considering that South Africa is home to 12 official languages, the surveys had to be administered in English due to the language ability of the researcher analyzing the collected data. However, the surveys were developed and administered considering the dialect used by the target population, adapting certain wording. The time and date were chosen carefully, to accommodate the students capacity of concentration. This means that the researcher visited the school one week before the training session started to administer the pre-survey during school hours on school grounds. The post-surveywas completed by the students one week after the last training session was conducted, again during school hours on the schools' premises. This ensured that the students were able to concentrate fully on the survey and had enough time to complete all items.

### Data analysis

The collected data was statistically analyzed using SPSS (version 29). Considering that this research is comparing two independent sample means for each survey item, the independent T-Test applied. The statistical significance was defined as *p* ≤ 0.05 (two-tailed), consistent with conventional practice in sport and sport for development research. This threshold reflects a 5% acceptable probability of Type I error and was established prior to analysis. Effect sizes were calculated using Cohen's *d*, with values of 0.2, 0.5, and 0.8 indicating small, medium, and large effects respectively (58). Exact *p*-values and effect sizes are reported throughout to allow readers to evaluate the practical significance of findings.

### Reflexivity

The discussion of results incorporates researcher reflexivity, acknowledging that the study was conducted in a Global South context by a Global North researcher. While reflexivity is commonly associated with qualitative research, the specific context of this study necessitated a critical examination of linguistic, cultural, and power dynamics, as emphasized by Mwaanga (30) and Scott & Soares Moura (2). To explore how the researcher's ethnicity, including linguistic, cultural, and ancestral background, shaped relationships, training sessions, and data collection, review sheets were completed by the researcher after each SFD session. These sheets documented successes, challenges, and particularly the dynamics between the researcher and participants. Such observations were instrumental in assessing the researcher's adaptability to the participants and their local environment.

### Concept development

**Figure 1:**
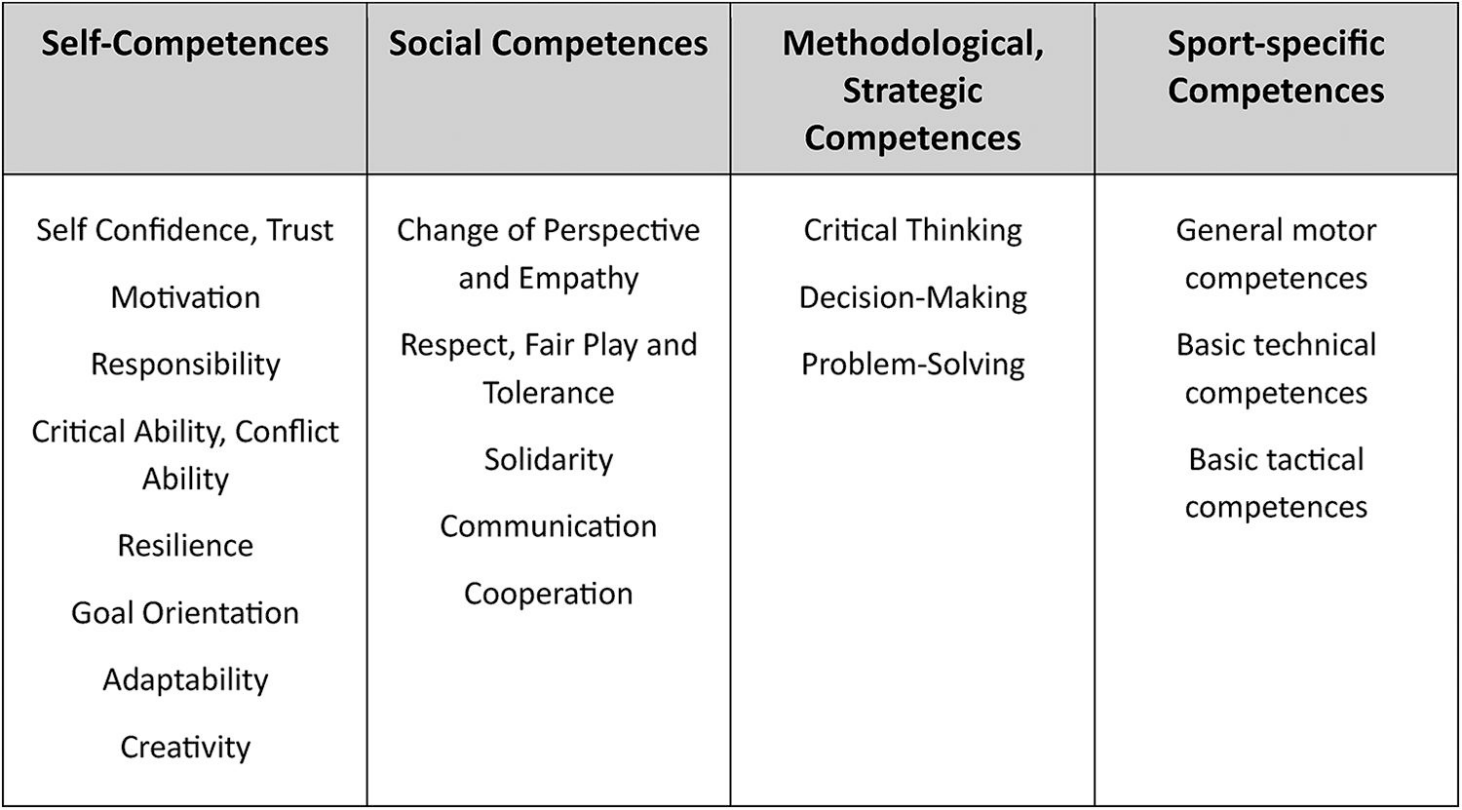
SFD competence clusters (37).

The development of competences, also known as life skills (31), is the core mechanism of the SFD model designed by the GIZ and the GSU. Competences are set out to enable children and young people to master the realities they face on a daily basis. The development of new competences or strengthening of already existing ones is therefore vital in tackling challenging life situations, particularly for youth who lack prosocial resources and support (32, 33). Competences can be categorized as either intrapersonal or interpersonal skills, and if developed and improved, can contribute to the achievement of development objectives, for example, in areas such as violence prevention or health (34, 35). The idea is therefore to develop competences amongst participants deemed as important in relation to specific development topics, in this case being ocean conservation. Grounded in the concept of the GIZ and GSU, competences are divided into four clusters, namely self, social, methodological, and sport specific competences (36). Grounded on the concept of ocean literacy, self and methodological competences are concerned with intrapersonal development, meaning the personal ability to reflect on challenges the ocean is facing and what ocean conservation constitutes. Social competences, on the other hand, refer to the interaction with others, targeting interpersonal development. In terms of ocean literacy, interpersonal competences would refer to the ability to communicate to others about ocean conservation. Sport specific competences are related directly to the physical development of motor, technical, and tactical competences of the respective activity, however, are not specifically connected to behavioral change. Each cluster of self, social and methodological competences contain a number of specific competences that can be attained via a SFD program (see Figure 1 for each cluster and associated competences). To understand the adoption and change of competences on an individual level, each competence is further divided into three levels: recognizing, assessing, acting^4^ (55). The level of recognizing refers to the attainment of knowledge about certain topics and the resources it stems from. Assessing is concerned with the ability to categorize the acquired knowledge and reflect on it critically to make informed decisions. The final level of acting presents the change of behavior in other life contexts. The levels of recognizing and assessing are directly connected to the development of self- and methodological competences, while acting refers to social competences, as their behavior ultimately impacts others. To successfully develop a competence, participants must attain all three levels. As competences take on different forms depending on the situation they are practiced within, it is paramount to adapt each competence to the specific context or anticipated development outcome of the SFD program. As described by the Experiential Learning Theory (4), this means that the chosen physical activities should create experiences that teach the participants to apply competences that align with the overall goal of the program, in this case ocean conservation related competences. Finally, these competences are conveyed and practiced within specifically developed SFD training session activities, taking ocean literacy and the Experiential Learning Theory into account.

### Ocean conservation related competences

Based on the topic of ocean conservation, the before mentioned newly developed competence framework is based on the objectives of the OGW and uses physical activities to teach competences that encourage young adolescents to engage in ocean conservation practices. Adapted to the OGW, and considering this study, the new framework only comprised of five competences deemed as essential to foster ocean conservation practices, which include responsibility, goal orientation, communication, critical thinking, and decision-making. Together with IAW, these competences were carefully selected by focusing on at least one competence from each cluster. Responsibility and Goal Orientation belonging to the cluster of self-competences, communication being part of the cluster of social competences, and critical thinking and decision-making being part of the methodological cluster. The sport activities used throughout the sessions additionally considered sport specific competences belonging to the fourth cluster, however, were not further considered regarding the analysis of behavioral change. To continue, the chosen competences were further developed according to the three levels of recognizing, assessing and acting, as previously described. In combination, the five competences chosen for ocean conservation SFD sessions were designed to equip participants with the necessary tools to lead a more ocean friendly lifestyle on a daily basis, however, taking into account their personal, social and cultural context. That is, understanding the resources and capacities the participants have at their disposal in their common environment. This particularly concerned the level of acting, making sure participants are truly able to practice their newly acquired competence in a safe and realistic manner.

### The competence reflection

For competences to be successfully attained, the ability to transfer them to other life contexts is of utmost importance and requires appropriate facilitation (38). Particular emphasis is therefore placed on the reflection part in each SFD training session, in which participants are encouraged to reflect on the experience they have had during the activity and reapply it to experiences they have already had in other life situations. The reflection is what differentiates a SFD training session from a regular sport session and is therefore a crucial component in the realization of development objectives (39). As emphasized by the Experiential Learning Theory (4), the reflection step is crucial to go from experience to adapted behavior. To facilitate an adequate reflection, each competence received specific reflection questions that targeted the understanding of the experience, connect it to other life contexts and finally encouraged the participants to think of practical solutions^5^.

### SFD training sessions

To continue, the above chosen ocean conservation related competences form the basis of the ongoing SFD training sessions, adapting the activities to facilitate their development, carefully considering the levels of recognizing, assessing and acting, and the reflection step. The SFD training sessions were each one hour long per group and were conducted once a week for four consecutive weeks. Tailored towards the content touched upon in the OGW, the SFD training sessions concentrated on the areas of marine pollution, overfishing, climate change, and marine protected areas, specifically addressing competences that are required to positively impact these ocean related challenges. The training sessions consisted of a warm-up activity, a main part and a cool down (40). All three sporting activities were designed to place the participants into a situation that required them to use a certain competence to achieve the goal of the activity. The activities were extracted from a number of SFD manuals developed by the GIZ^6^ and were adapted to fit the context and topic of the session, as well as the environmental conditions and resources available. Together with the developed reflection questions, the competences built the foundation of the training sessions, guiding the activities and the overall reflection towards the anticipated outcome.

The sporting activities within this conceptual framework were chosen to fit into the SFD framework introduced by Coalter (9), referring to the *plus sport* approach^7^. In the case of this SFD conceptual framework for ocean conservation, the *plus sport* approach means that sport and physical activities are used to convey ocean conservation messages and develop relevant competences amongst adolescents. While there is still an increased focus on sport specific competences, that foster motor, technical and tactical skills, as described in the competence clusters, the framework is mainly designed to convey competences that participants require to successfully engage in ocean conservation practices. The chosen activities always included some form of running, jumping, dribbling with balls, agility as well as sport specific strategic exercises. However, competitive sport activities were most popular amongst the participants, suggesting that competitive sports seemed to be most engaging. Going forward, the activities included a competitive element to keep the participants interested. Each session was framed with an ocean conservation related topic (e.g., overfishing, meaning that all activities were adapted to fit this theme). The sport activities included games such as chain catching during which one chosen player was trying to catch all other players on the field. Once a player was caught, they started to hold hands with the catcher and began to form a chain with which they continued catching the others. This continues until all players are part of the chain. Under the theme of overfishing, the activity was framed as fisheries catching all the fish in the ocean until none were left. Through the activity, the participants physically went through the catching process and experienced how quickly all players became part of the chain. The reflection afterward further contextualized this by linking the loss of players on the field to the loss of fish in the ocean and the group continued to discuss how this affected their own lives on a daily basis.

Each activity was carefully designed to improve their physical abilities while considering which activities are best suited to foster competences such as responsibility and further transferring these to the topic of ocean conservation. This process is rather complex and requires careful triangulation of fostering physical and individual competences with ocean conservation related competences.

## Results

A total of 67 participants completed the pre-survey and 64 participants completed the post survey^8^. To begin with, the participants were asked to identify the correct competences regarding specific actions. The only significant difference, t (123.9) = 2.083, *p* = 0.039, d = 0.37, was found when asking the participants what competence would be required when “*explaining to others what they can do to keep the ocean clean*”, indicating a small-to-medium effect. However, the responses developed negatively, as the number of participants correctly identifying the competence *Communication* dropped from 77.6% in the pre-survey (M = 0.78, SD = 0.42) to 60.9% in the post-survey (M = 0.61, SD = 0.49). No significant differences were found for all other items regarding the identification of competences as shown in Table 1. Nevertheless, comparing the frequency distributions, one can see that a high percentage of participants were already able to correctly identify the competences of each item in the pre-survey confidently. When asked what competence was required when “*separating your own rubbish*”, 89.6% correctly identified *Responsibility* in the pre-survey compared to 92.2% in the post survey. Further, participants were asked what competence would constitute “*wondering about your own impact on the ocean*” and the number of participants correctly identifying *Critical Thinking* dropped from 82.1% in the pre-survey to 73.4% in the post survey. Continuing, participants were asked what competences would be necessary for “*planning to use less plastic*” and the number of participants correctly identifying *Goal orientation* increased from 32.8% to 45.3%. Lastly, item 19 asked the participants what competence would be “*doing things to help keep the ocean clean*” of which the number of participants correctly identifying the competence *Responsibility* decreased from 76.1% to 67.2%.

**Table 1:** SFD Pre vs. Post-survey Competence based Questions.

**Q: Separating your own rubbish into the correct dustbin is an example of…**					
**Pre-Survey**		Post Survey			
***M***	***SD***	***M***	***SD***	***t-value & p-value***	***Cohens’ d***
**.89**	.31	.92	.27	t (129) = −.519, *p* = .605	***d*** **=** **0.10**;*small effect*
**Q: Explaining to others what they can do to keep the ocean clean is an example of…**					
**Pre-Survey**		Post Survey			
***M***	***SD***	***M***	***SD***	***t-value & p-value***	***Cohens’ d***
**.78**	.42	.61	.49	t (123.9) = 2.083, *p* = .039*	*d* = **0.37**; *small-to-medium effect*
**Q: Wondering about your impact on the ocean is an example of…**					
**Pre-Survey**		Post Survey			
***M***	***SD***	***M***	***SD***	***t-value & p-value***	***Cohens’ d***
**.82**	.39	.73	.45	t (124.7) = 1.186, *p* = .2.38	***d*** **=** **0.21**; *small effect*
**Q: Planning to use less plastic packaging at home is an example of…**					
**Pre-Survey**		Post Survey			
***M***	***SD***	***M***	***SD***	***t-value & p-value***	***Cohens’ d***
**.33**	.47	.45	.50	t (127.6) = −1.463, *p* = .146	***d*** **=** **0.25**; *small effect*
**Q: Doing things to help keep the ocean clean is an example of…**					
**Pre-Survey**		Post Survey			
***M***	***SD***	***M***	***SD***	***t-value & p-value***	***Cohens’ d***
**.77**	.42	.69	.46	t (122.9) = 1.007, *p* = .316	***d*** **=** **0.18**; *small effect*

*Statistically significant.

To continue, participants were asked about their own ocean conservation related actions. Thereby, no statistically significant differences were found across any of the remaining four items as can be obtained from Table 2. Looking at the frequency distribution, however, some changes can be observed. For example, participants were first asked to “*explain their reaction when they would see someone throwing rubbish into the see*”. The results indicated a shift in competences used, while *Confrontation* decreased from 74.6% to 65.6% and *Responsibility* increased from 17.9% to 20.3%.

**Table 2:** SFD Pre vs. Post-survey Competence based Questions.

**Q: You see someone throwing rubbish into the ocean. How do you react?**					
**Pre-Survey**		Post Survey			
***M***	***SD***	***M***	***SD***	***t-value & p-value***	***Cohens’ d***
**2.00**	.82	1.9	.69	t (124) = .751, *p* = .454	***d*** **=** **0.13**; *small effect*
**Q: When you go shopping for food you can either buy a new plastic bag or bring a bag that you already have. Which one do you choose?**					
**Pre-Survey**		Post Survey			
***M***	***SD***	***M***	***SD***	***t-value & p-value***	***Cohens’ d***
**1.17**	.38	1.13	.34	t (124) = .593, *p* = .554	***d*** **=** **0.11**; *small effect*
**Q: You spend a day at the beach with your family and you bring food and drinks with you. When are leaving you have a big bag full of rubbish. What do you do with it?**					
**Pre-Survey**		Post Survey			
***M***	***SD***	***M***	***SD***	***t-value & p-value***	***Cohens’ d***
**1.75**	.47	1.78	.42	t (120) = −.318, *p* = .751	***d*** **=** **0.07;** *negligible effect*
**Q: You have an empty plastic cup from your juice. What dustbin do you throw it in?**					
**Pre-Survey**		Post Survey			
***M***	***SD***	***M***	***SD***	***t-value & p-value***	***Cohens’ d***
**1.08**	.27	1.04	.19	t (121) = .967, *p* = .336	***d*** **=** **0.17**; *small effect*

Continuing, the participants were asked “*if they would bring an old bag with them to go shopping or rather buy a new one*”. The number of participants that would bring an old bag remained relatively constant from pre to post survey, increasing slightly from 80.6% to 82.8%. The number of participants that would buy a new bag decreased from 16.4% to 12.5%. Further, participants were asked “*what they would do with their own rubbish when they spend a day at the beach*”*.* Participants that would take rubbish from the beach to the bin at home decreased from 25.4% in the pre-survey to 20.3% in the post survey, while the number of participants taking the rubbish to a bin nearby increased from 68.7% to 70.3%. Nevertheless, no participant indicated in the post-surveythat they would leave rubbish on the beach, compared to 1.5% in the pre-survey. Lastly, participants were asked “*in what bin a plastic cup belongs*”. The participants correctly identifying the recycling bin decreased from 91% to 85.9%, while the number of participants that would throw it into a regular bin also decreased from 7.5% to 3.1%. However, the number of participants not giving any response for these questions increased from 1.5% to 10.9%, indicating a knowledge gap.

Lastly, participants were encouraged to assess their own level of competences. The responses presented no significant difference for any of the included items. For the means, standard deviation, as well as t and *p* values see Table 3. However, changes could be observed in the frequencies of responses given, displaying a slightly negative trend. Firstly, participants were asked if they believe that “*what others do does not concern them*”. The number of participants disagreeing with that statement decreased from 41.8% to 29.7%, while the participants that were unsure increased from 25.4% to 35.9%. Further, participants were asked whether “*it is easier to make changes when setting goals*” and the number of participants agreeing with this statement dropped from 89.6% to 85.9%. To continue, the participants were asked whether they “*feel comfortable speaking up to their friends*”. Again, the number of participants agreeing with that statement decreased from 67.2% in the pre-survey to 59.4% in the post survey, while the number of participants disagreeing increased from 22.4% to 29.7%. Similarly, the number of participants “*feeling that they could encourage others to help protect the ocean*” decreased from 88.1% to 76.6% in the post survey, while the number of participants disagreeing with that statement increased from 1.5% to 6.3%. Further, participants were asked “*if they often decide things based on what their friends are doing*”*.* Hereby, the number of participants agreeing with this statement increased from 9% to 14.1%, while the number of participants disagreeing dropped from 73.1% to 65.6%. Lastly, the participants were asked if they “*know where to find information about the ocean*” and the number of participants agreeing increased from 71.6% to 73.4%. Overall, a slight change in assessment patterns can be observed.

**Table 3:** SFD Pre vs. Post-survey Competence based Questions.

**Q: What other people are doing does not concern me.**					
**Pre-Survey**		Post Survey			
***M***	***SD***	*M*	***SD***	***t-value & p-value***	***Cohens’ d***
**1.93**	.77	2.03	.84	t (128) = −.755, *p* = .452	***d*** **=** **0.12;** *small effect*
**Q: It is easier to make changes if you set specific goals.**					
**Pre-Survey**		Post Survey			
***M***	***SD***	***M***	***SD***	***t-value & p-value***	
**1.16**	.51	1.18	.53	t (127) = −.145, *p* = .885	***d*** **=** **0.04;** *negligible effect*
**Q: I feel comfortable saying something to my friends if they are doing something that I disagree with.**					
**Pre-Survey**		Post Survey			
***M***	***SD***	***M***	***SD***	***t-value & p-value***	***Cohens’ d***
**1.43**	.68	1.47	.65	t (127) = −.299, *p* = .766	***d*** **=** **0.06;** *negligible effect*
**Q: I feel that I can encourage others to help protect the ocean.**					
**Pre-Survey**		Post Survey			
***M***	***SD***	***M***	***SD***	***t-value & p-value***	***Cohens’ d***
**1.22**	.62	1.33	.70	t (126) = −.889, *p* = .376	***d*** **=** **0.17;** *small effect*
**Q: I often decide things based on what my friends are doing.**					
**Pre-Survey**		Post Survey			
***M***	***SD***	***M***	***SD***	***t-value & p-value***	***Cohens’ d***
**2.09**	.51	2.05	.58	t (128) = .437, *p* = .663	***d*** **=** **0.07;** *negligible effect*
**Q: I know where to find information about the ocean.**					
**Pre-Survey**		Post Survey			
***M***	***SD***	***M***	***SD***	***t-value & p-value***	***Cohens’ d***
**1.47**	.80	1.43	.78	t (128) = .353, *p* = .725	***d*** **=** **0.05;** *negligible effect*

## Discussion

The aim of this research was to identify whether a SFD program is able to facilitate the development of ocean conservation related competences amongst young adolescents in Cape Town, South Africa, encouraging the embodiment of environmental protection related behaviour.

While the only statistically significant result revealed a decline in participants' ability to correctly identify the competence Communication, dropping from 77.6% in the pre-survey to 60.9% in the post-survey, the remaining results indicate no further statistically significant changes across competence development, behavioural response, or self-assessed competence items. However, interpreting these findings requires looking beyond the t-test results alone. Examining the frequency distributions reveals that a considerable proportion of participants already demonstrated relatively high levels of awareness and intended behaviorprior to the intervention, suggesting a limited the scope for statistically detectable improvement. For example, 89.6% of participants correctly identified Responsibility as the competence underpinning waste separation in the pre-survey, and 91% correctly identified the appropriate bin for a plastic cup. Where baseline scores are already high, the absence of significant change should not be confused with the absence of impact. Furthermore, subtle but noteworthy shifts were observable in the frequency distributions across behavioural items, such as no participant indicating they would leave rubbish on the beach in the post-survey compared to 1.5% in the pre-survey, pointing to small but meaningful changes that aggregate statistics alone fail to capture.

Perhaps the most theoretically interesting pattern is the slight negative trend observed in participants' self-assessed competences. The proportion of participants feeling able to encourage others to protect the ocean dropped from 88.1% to 76.6%, and those feeling comfortable speaking up to friends regarding environmentally harmful behaviordecreased from 67.2% to 59.4%. Rather than suggesting a deterioration of competences, this development may reflect a process of critical self-awareness fostered through the experiential learning cycle (4). As participants engaged with ocean conservation concepts through embodied, reflective activities, they may have developed a more nuanced and realistic appraisal of their own competences, temporarily reassessing themselves more critically as their understanding deepened. One could interpret that the participants understood more in-depth what the required competences are and what they look like, and therefore assessed themselves more critically towards acquiring them. However, this negative trend in self-assessment is mirrored by the study's only statistically significant finding, the decline in correctly identifying Communication as a competence. Together, these patterns present a consistency, as rather than strengthening participants' competence identification and self-assessed ability, the intervention appears to have initiated a critical re-evaluation of both. While the proportion of participants feeling they could encourage others to protect the ocean highlights a slight increase from pre- to post-survey (M = 1.22 to M = 1.33, d = 0.17), this result did not reach statistical significance and should be interpreted with caution. These findings point to the value of longitudinal research in capturing outcomes that a four-week pilot study, by its very nature, is unable to fully realise, suggesting that future studies would benefit from extended programme durations and follow-up measurements to assess whether initial shifts in critical self-awareness translate into sustained competence development and behavioural change over time.

As described throughout the concept development, the SFD training sessions consisted of different activities which required the participants to use certain competences to achieve a goal. For example, a set of behavior was described to the participants and for one team to win the activity, the participants had to critically reflect on that behavior as either positively or negatively impacting ocean conservation and then run towards a cone that symbolized the correct answer, relating this exercise to the competence of critical thinking. To deepen the impact of the activity, a reflection took place afterwards at which participants were asked specific questions about the content of the activity and how this relates to other life contexts. However, the negative results of the survey lead to the conclusion that the competences were not adequately addressed throughout the activity or the reflection part. It seems that the participants did not comprehend which competences were the subject of the training sessions and more importantly, what they look like regarding ocean conservation, suggesting that the sporting activities alone, as put by Kolb (4) are not sufficient to develop or strengthen competences. A much more rigorous and transparent approach is needed, where participants are taught what a competence means, what it includes and how it manifests in their own behavior. Each competence should be clearly named throughout the activities, defined and then reflected upon, relating it to other life contexts. Placing the participants in situations that require them to use a certain competence, does not mean they understand that they are applying it, let alone how this refers to their future behavior. Therefore, one can conclude that the reflections were not able to bridge the transfer from pitch to other life contexts, making participants understand the competence they used and why this is important for ocean conservation. Additionally, it can be assumed that the choice of location has a profound impact on the comprehension of certain topics, especially ocean conservation. The training sessions took place on the school grounds instead of the beach, possibly making the topic of ocean conservation too abstract for the participants. While the location made it possible to use more traditional forms of physical activities such as running or dribbling with balls, the actual bodily contact with the ocean could provide a greater attachment and understanding of the impact their behavior has on the ocean, as suggested in the study by Olive (24). The ocean is tangible, and the challenges it faces can be seen, embodied and felt physically by the participants, making it particularly useful to be right at the beach when learning about ocean conservation. It creates a sense of embodied connection and impact on oneself. Particularly when this location is considered as your home area, as put forward by Lucrezi and du Plessis (26). Therefore, one could claim that the location in which an experience takes place, is just as important to foster competences as the experience itself, particularly in terms of the natural environment. However, this would mean that the Experiential Learning Theory (4) should be extended, placing greater emphasis on the location in which the experience is generated. Further research would be necessary to prove this claim, though regarding this study it appears to be a valid factor, considering the difference of being on land and being in or at the ocean during an activity.

Further, analyzing the collected results, it becomes clear that the uptake of ocean conservation related competences is subject to more externalities than initially anticipated. Considering that the researcher was visiting Cape Town, South Africa, coming from the European country of Germany, situated in the so-called Global North, a great deal of reflexivity is required (15, 16). Although this form of bias reflection is mainly practiced within qualitative research, particularly in ethnographic studies (41), the context of the implementation of the SFD conceptual framework by a Global North researcher in a community of the Global South basically dictates an in-depth reflection on the researcher's part. Additionally, after closer inspection, it became clear that those cultural differences did have an impact on the process of the SFD training sessions as well as the gathered results. The following reflection additionally pertains to the limitations of the study overall.

Firstly, the most prominent factor that had to be considered was the difference in languages. South Africa is home to 12 official languages since 2018, including one South African sign language (42). Through personal observations and exchanges with the local IAW coaches, the participants are said to speak a set of many different languages in their communities. The languages of Zulu, Xhosa, Afrikaans and English only display a small number of predominantly spoken languages in the participants homes. The variety of languages remaining in their communities demonstrate the struggles deeply rooted within their history, exemplifying the continuous changes in power within the country through white colonization and oppression of the majority of black and colored communities and their native languages (42). Consequently, language was a very important point to consider in the development of the survey items, as well as the communication during the SFD training sessions. Considering that the researcher only speaks English and German, there was a predetermined dominance asserted (43, 44), in which the participants had to answer the survey in English to be analyzed by the researcher, regardless of whether this was their first spoken language or not. While the survey tried to include the daily wording used by the target population, such as “dustbin” instead of “bin”, the chosen language could have led to certain questions not being understood properly by the participants, already skewing results (45). Although the survey items were all read out loud by the researcher when the surveys were being completed, it does not guarantee that all participants understood what was asked of them. This issue certainly carries the notion of a Global North dominance, expecting participants to adapt to the researcher's identity, and should be considered in future research endeavors.

To continue, the process of the delivery of the training sessions was subject to change as one training session had to be rescheduled to take place on a Friday morning. The reason being that many participants do not visit the school on Fridays due to religious customs, leading to a much smaller participant group on that day. While the smaller group did have a positive effect on the delivery of the training session, these cultural and religious customs should be considered before the implementation of the session to give all participants equal possibilities to join. This point makes undoubtably clear that the researcher was not familiar with the cultural context of the participants, pointing towards the researcher being positioned rather outside of the participants communities and traditions (41). It brings forward the naïve presumption of a foreign researcher allegedly knowing how to engage in ocean conservation in a country they are not familiar with.

Further, it is highly important to address that despite the foreign nature of the researcher to the country including its communities and local traditions and customs, the access to the participants through the OGW of IAW and the school was incredibly easy. The NGO IAW has a very longstanding and positive relationship to the participating school, marking their request to participate in this research study with previous developed trust. However, while there was an IAW coach present at each training session, it should be reflected that the researcher was still able to teach the participants without any interference. This carries the notion that the researcher from the Global North is omniscient and trustworthy despite their lack in awareness of the South African context explained above. It becomes clear that the power asymmetry developed through history of colonization is deeply rooted in general society and continuously reproduced (46). Particularly noteworthy is the inability of the researcher to further engage with the participants of the sessions outside of the school, clearly impacting their relationship and the concept of the researcher being an outsider (41). Additionally, the length of the entire SFD training sessions was about four consecutive weeks, with training sessions taking place once a week, one hour per group. Considering that this program was set up as a pilot, the restricted timeframe was clearly not sufficient to teach competences in a sustainable manner. Despite being a pilot study, this further highlights the persisting issue of dominance of the researcher over the study subjects. While the researcher can simply come and conduct research depending on their own time and resources, the participants were subject to this study design regardless of whether this would be useful to them or not.

The reflexivity elements discussed above, and the overall insignificant results of this study additionally put the SFD model, as it was applied and as it is often practiced within the sector, into question. Born out of colonialism, the SFD model has been rightfully criticized of reproducing neocolonial power structures (47, 48), which was made undoubtably clear throughout this study. Stretching all the way from language to the development of the model and the analysis of the data, the knowledge applied and reproduced throughout this conceptual framework was predominantly Global North dominated and although the topics and the content of the sessions were adapted to fit the local OGW, the objectives to be achieved were still impacted by the researcher's idea of what successful ocean conservation looks like. Perhaps the language of sport is not as universal as it has always been perceived to be but depends on cultural meanings and interpretations of the local target population. It seems to be dependent on their perception of sport, the meaning it has for them and ultimately how they interpret the messages they receive or gain by taking part in that sport. If the SFD model does not take this into account and is only designed out of one perspective (in this case Global North), it clearly demonstrates hegemonic power relations and influences whether the outcome will be successful. The model should have been centered around the local issues to begin with, asking the right questions as to what the participants require to engage in ocean conservation rather than implementing a predeveloped concept. While it was assumed that the SFD model was adapted to fit the context and specifically the lack of resources available, the model itself does little to overcome these already existing challenges. Even if the participants would have successfully developed competences, the lack of resources would remain. In other words, teaching young adolescents about how to take care of the oceans resources while already dealing with scarce resources as it is, seems paradox and quite frankly perpetuates the unequal resource allocation globally by teaching them to cope with this situation rather than changing it. One issue comes to mind, considering that since 1995 physical education has not been a stand-alone subject in South Africa but rather absorbed in other subjects (49), the SFD model seems particularly displaced within this context. In fact, it further draws attention to the lack of sports equipment and adequate sporting grounds within the school. The sessions would have potentially generated more impact if taking place right at the ocean to enable the embodied experience, much like the OGW is conducted. After going through the OGW the participants possibly associate this area with physical activity more than their own school grounds where no sport lessons seem to take place otherwise. This suggests that for the SFD model to find the necessary traction to achieve its objectives, it is important to adapt it to the way sport and physical activity is already practiced and experienced within the target community, considering movements and sports most played, while choosing a suitable environment that compliments the activities and overall goals.

This study reveals that sport itself is being continuously used to address challenges that are way beyond the sporting grounds in areas that are not suitable. Asking the SFD model to foster individual competences is one thing but to tackle issues that are fueled by global power disbalances such as climate change, might be illusional.

Lastly, it should be critically assessed whether the use of a quantitative method was appropriate in this context overall. Although quantitative research is said to produce replicable results and should exclude a certain type of bias, the reflexivity shows that there was still a great influence on the results, even when collecting numerical data. In retrospect, it can be contested that the development of intra- and interpersonal competences can or even should be analyzed numerically. While the results have proven to be statistically insignificant since the participants were not able to specifically name the competences that they utilized throughout the training sessions in the survey, they might have been able to subconsciously understand them and place them into context. A qualitative research approach could have given greater opportunity for the participants to demonstrate their change of behavior and detect whether they grasped the concept of ocean conservation without naming specific terminology. As previously explained, competences are set out to influence behavior and while ocean conservation requires a certain level of prior knowledge, it is the behavior that is the important factor, regardless of the ability to name a competence by its predetermined terminology. Possibly the terminology used by the participants could simply differ due to language differences, as previously discussed. Concluding, the idea that a Global North researcher can numerically analyze intrinsic competence development, once again refers to the asymmetric power dynamics (2). It predetermines that there is a standard level of competence acquisition to be aspired to, which was set out by the researcher. This article assesses this own choice of methodology very critically and calls for future research to highly consider this notion and therefore suggesting a qualitative approach.

Considering the insignificant results of the study and the analysis of the reflexivity, it becomes clear that intrinsic competences should be taught and reflected upon in a more local context, taking into account the participants cultural and historical background and how this impacts their understanding of these competences. A power asymmetry in knowledge was asserted, setting a standard regarding what is perceived as a competence, how it manifests in behavior and finally, how it should be measured. Particularly the quantitative measurement is set by the researcher's standards, and coming from the Global North, these standards might not necessarily align with how competences should be identified and measured in the South African context. This particularly questions the validity and necessity of applying this SFD conceptual framework. That a foreign researcher is able to understand how climate change impacts the ocean in South Africa and knows how competences manifest to mitigate those challenges is a presumption at best.

The findings and reflexivity presented throughout this discussion point towards several important directions for future research. Firstly, a longitudinal study design would be particularly valuable in determining whether the critical self-awareness and subtle shifts in behaviorobserved in this pilot study develop into sustained competence acquisition and ocean conservation behaviorover time. A single pre- and post-survey measurement, as employed here, is inherently limited in its ability to capture the gradual and cumulative nature of the experiential learning cycle (4). Secondly, future research would benefit greatly from adopting a qualitative or mixed-methods approach, allowing participants to demonstrate competence development in their own words and cultural framings, rather than through predetermined terminology set by an outside researcher. This would not only produce richer and more contextually valid data but would also begin to address the power asymmetry that quantitative measurement inevitably reproduces in this context. Thirdly, and perhaps most critically, future studies should be co-designed with members of the local community from the outset, not merely adapted to fit the local context after the fact. This would ensure that the competences being taught, the methods being used, and the outcomes being measured are genuinely relevant and meaningful to the population in question, rather than reflecting the assumptions of a Global North researcher. Finally, replicating this study in a coastal setting, with activities taking place at or in the ocean rather than on school grounds, would allow researchers to assess the extent to which embodied environmental experience shapes competence development and behavioural change in ways that land-based activities cannot.

## Conclusion

The challenges the ocean is facing today, are increasingly impacting the global population and have dire consequences for coastal communities in South Africa (20). While international standards are being developed, a substantial gap in ocean related knowledge amongst the public and a lack of tools to adverse the effects of climate change remain. Although, the developed SFD conceptual framework is a first indication of how ocean conservation could be incorporated in a SFD program to bridge this gap, it has become undoubtably clear that the delivery and success of such programs depend on local intricacies (14, 50). Differing contexts can impact behavior and the development of competences, considering that populations around the globe experience issues such as climate change differently. Taking into account other hardships such as socio-economic challenges, each population will require a very locally centralized approach to teaching competences, considering the resources they have available. Particularly the challenges the ocean is facing, have different effects on the population depending on the location, which ultimately influences how populations engage with the topic and further, what they have to do to protect themselves.

The findings of this study generate several concrete recommendations for the further development of the SFD conceptual framework. The framework should be redesigned as a collaborative process, co-created with members of the local community who fully understand the cultural, linguistic, and socio-economic specificities of the target population. This would not only improve the relevance and effectiveness of the programme but would work towards balancing the Global North-South power dynamics that this study has critically exposed. Furthermore, each competence addressed throughout the training sessions should be explicitly named, defined, and reflected upon in relation to participants' own lived experiences and local contexts, rather than assumed to be absorbed through activity alone. The framework should additionally prioritise the location of delivery as a core design element, bringing participants into direct embodied contact with the ocean wherever possible, as the immersive experience of the natural environment appears to be a key condition for meaningful engagement with ocean conservation. As a foreign researcher one should therefore carefully consider whether they are the right person to conduct such programs and associated research, or whether this should be delivered by someone from the local community who fully understands the local specificities.

The results of this study clearly highlight that the experience, as laid out by Kolb (4) in the Experiential Learning Theory, cannot be underestimated, particularly regarding issues of the natural environment. The sporting activities on the school grounds may have helped to retain knowledge but it did not bring the issue of ocean conservation to the forefront. It leads to the assumption that the embodied experience with the ocean places much greater emphasis on its wonders but also its existing threats, due to the immersive experience one can have. The direct experience is therefore a key element to a change of behavior. The short-term nature of this pilot study does suggest building on this research to develop a longer SFD program that could more sustainably teach ocean conservation related competences. Future programs should explore a different set of competences while adjusting the training sessions with activities that are culturally resonant and adapted to the local language and community context.

The persisting challenges increasingly require efforts to protect the ocean and the communities that depend on it. Continued research about the right methods and tools is therefore indispensable. A longitudinal design could indicate whether SFD can in some form play a role in mitigating these challenges in the future, but for it to do so meaningfully, it must begin by listening to the communities it seeks to serve.

## Data Availability

The original contributions presented in the study are included in the article/Supplementary Material, further inquiries can be directed to the corresponding author.

## Footnotes

^1^It is acknowledged that a number of abbreviations exist, referring to the term Sport for Development. However, for the purpose of this research, this article will continue with the abbreviation SFD.

^2^The concept of SFD by the GIZ and GSU was a result of the German Federal Ministry (BMZ) commissioning the GIZ to implement SFD in their global development projects to contribute to the UN SDGs.

^3^The Safeguarding Practitioner Guidelines was developed by the African Union Sports Council (AUSC) and the German Agency for International Cooperation (GIZ).

^4^This structure was adapted from the Global Learning model in the Curriculum Framework Education for Sustainable Development, developed by the Standing Conference of the German Ministers of Education and Culture (KMK) and BMZ in 2016.

^5^The reflect, connect, apply method in the reflection was adapted from the EDU:PACT Network from Right to Play in 2019.

^6^These manuals were accessed via the Sport for Development Resource Toolkit, a webpage developed and designed in cooperation with the German Sport University Cologne as part of the commission by the BMZ. As of January 2026, this website is hosted by the S4D Network Germany.

^7^The sport plus and plus sport approach differentiate in their intended focus. While the sport plus approach is primarily focused on the delivery of sporting activities and the increase of participation which adds to the achievement of non-sport related outcomes, the plus sport approach has social change and the development of non-sporting outcomes at the heart of its concept, using sport as tool to achieve these.

^8^The statistical abbreviations used throughout the results are as follows: M=Mean Value (the average value from the data set); SD=Standard Deviation (how far or close the value is from the standard mean value); t value=difference between one average in a data set to another; p value=probability of the calculated value to be true and whether it is significant.
